# Design and Synthesis of Ranitidine Analogs as Multi-Target Directed Ligands for the Treatment of Alzheimer’s Disease

**DOI:** 10.3390/ijms22063120

**Published:** 2021-03-18

**Authors:** Jie Gao, Chen Suo, Jui-Heng Tseng, Melissa A. Moss, Alvin V. Terry, James Chapman

**Affiliations:** 1 Department of Clinical and Diagnostic Science, University of Alabama at Birmingham, Birmingham, AL 35294, USA; 2 Department of Chemical Engineering and Biomedical Engineering, University of South Carolina, Columbia, SC 29208, USA; suochen@crc.com.hk (C.S.); tsengj@neurology.unc.edu (J.-H.T.); mossme@cec.sc.edu (M.A.M.); 3 Department of Pharmacology and Toxicology, Augusta University, Augusta, GA 30912, USA; aterry@augusta.edu; 4 Department of Discovery and Biomedical Sciences, University of South Carolina, Columbia, SC 29208, USA; chapman@cop.sc.edu

**Keywords:** Alzheimer’s disease, amyloid β (Aβ) aggregation, acetylcholinesterase, multi-target directed ligands, naphthalimide, thioflavin T

## Abstract

The aggregation of amyloid β (Aβ) peptides and deposition of amyloid plaques are implicated in the pathogenesis of Alzheimer’s disease (AD). Therefore, blocking Aβ aggregation with small molecules has been proposed as one therapeutic approach for AD. In the present study, a series of ranitidine analogs containing cyclic imide isosteres were synthesized and their inhibitory activities toward Aβ aggregation were evaluated using in vitro thioflavin T assays. The structure–activity relationship revealed that the 1,8-naphthalimide moiety provided profound inhibition of Aβ aggregation and structural modifications on the other parts of the parent molecule (compound **6**) maintained similar efficacy. Some of these ranitidine analogs also possessed potent inhibitory activities of acetylcholinesterase (AChE), which is another therapeutic target in AD. These ranitidine analogs, by addressing both Aβ aggregation and AChE, offer insight into the key chemical features of a new type of multi-target directed ligands for the pharmaceutical treatment of AD.

## 1. Introduction

Alzheimer’s disease (AD) is the most common form of dementia in the elderly population. One of the main pathological hallmarks in AD is the extracellular amyloid β (Aβ) plaques, which deposit between neurons, disrupt neural functions, and contribute to neuronal death [1]. The Aβ plaques are formed by the aggregation of polymorphous Aβ monomers. In particular, Aβ_40_ and Aβ_42_ are prone to aggregate into various assemblies [2,3]. Therefore, the prevention of aggregation of these monomers is considered to be a promising therapeutic approach for AD therapy. 

The dipeptide Phe19-Phe20 in the Aβ monomeric structure has been reported to play a critical role in aggregation [4]. A derivative structure from this dipeptide, two aromatic end groups connected by a linker, has been proposed to act as an ideal scaffold for an Aβ aggregation inhibitor by disrupting the π-π stacking between Aβ monomers through interaction with the phenylalanine residues 19 and 20 [5]. Several molecules derived from this structure have shown potent inhibition of Aβ aggregation [6,7,8]. 

Most of the current pharmaceutical treatments for AD are acetylcholinesterase (AChE) inhibitors. Unfortunately, these currently approved drugs have been found to benefit only about half the individuals who take them and to only temporarily decrease symptoms [9]. It is now well recognized that AD is multifactorial in nature, therefore, Cavalli A. et al. [10] proposed “multi-target-directed ligands” (MTDLs) for the treatment of AD. MTDLs describe single compounds “that interact with the multiple targets thought to be responsible for disease pathogenesis” [10]. MTDLs might present a feasible avenue to provide optimal therapeutic effects in AD. 

**Figure 1:**
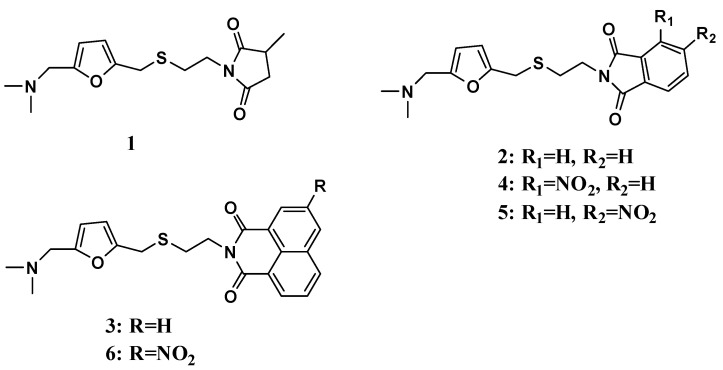
The structure of ranitidine analogs containing cyclic imide isosteres.

In one of our previous studies, a series of ranitidine analogs containing cyclic imide isosteres were synthesized as cognitive enhancers for the treatment of AD. A 3-nitro-1,8-naphthalimide derivative (compound **6**, Figure 1) was found to be a potent AChE inhibitor with IC_50_ = 0.15 µM. [11]. It is interesting that the structures of these ranitidine analogs conform to the proposed ideal scaffold of Aβ aggregation inhibitors. This study employed compound **6** as a prototypical compound for the development of a new series of compounds inhibiting both acetylcholinesterase and Aβ aggregation simultaneously as MTDLs for the treatment of AD. 

## 2. Results and Discussion

### 2.1. Aβ Monomer Aggregation Inhibition by Ranitidine Analogs Containing Cyclic Imide Isosteres

**Figure 2:**
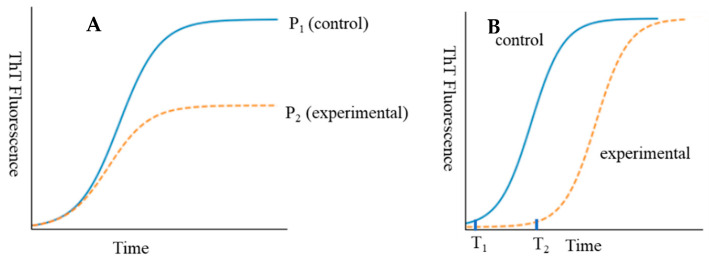
Assessment of the inhibition of Aβ monomer aggregation. Monomer aggregation assays were conducted in the absence (solid line, control) and presence (dotted line, experimental) of the ranitidine analogs, and aggregate formation was monitored as an increase in the ThT fluorescence. (**A**) Inhibition that decreases the quantity of aggregates formed at equilibrium is reported as the plateau reduction, the percentage decrease of the plateau ThT fluorescence in the presence of inhibitor, P2, compared to the plateau for the control, P1. (**B**) Inhibition that extends the time to aggregate formation is reported as the lag time extension. It is evidenced as an increase in the experimental lag time, T2, relative to the lag time for the control, T1, and evaluated as a fold increase relative to the control, T2/T1.

Synthetic Aβ_40_ was used in this study because it is the predominant isoform found in amyloid plaques isolated from AD brain [12]. Thioflavin T (ThT), a fluorescent compound that binds specifically to amyloid β-sheet structures to yield a shifted, enhanced florescence, was used to monitor the appearance of aggregates. Aggregation of Aβ monomer was observed to follow the expected trend, including a lag time to aggregate formation, aggregate growth, and an equilibrium plateau (Figure 1, control). The inhibitory effect of compounds on Aβ aggregation was evidenced as a reduction in the equilibrium plateau and/or an increase in the lag time [13]. The plateau reduction (Figure 2A), which indicates inhibition that reduces the extent of Aβ aggregation, was defined as the percentage decrease of the plateau ThT fluorescence in the presence of inhibitor, P2, compared to the plateau ThT fluorescence in the absence of inhibitor, P1. The lag time extension (Figure 2B), which indicates inhibition during the early stages of Aβ aggregation, was determined as a fold increase of the time at which ThT fluorescence is first observed in the presence vs. absence of inhibitor, T2 and T1, respectively. 

As shown in Table 1, all ranitidine analogs containing cyclic imide isosteres have an aromatic furan group at one end of their structures, while the number of aromatic rings at the other end of structures vary from 0–2. The plateau reductions by compounds **1**–**3** were improved as the number of fused unsubstituted aromatic rings increased on the cyclic imide moiety. Compound 3 with a 1,8-naphthalimide group presented a substantial plateau reduction of 60.9% ± 5.7%. This result preliminarily indicates that pronounced plateau reduction of Aβ aggregation might require at least two aromatic rings fused to the cyclic imide. The addition of a nitro group on an aromatic ring might be able to enhance the plateau reduction, as evidenced by compound 5 vs. 2 as well as compound 6 vs. 3. It should be noted that the position of the nitro group on the aromatic ring might be critical since the 2-nitro substitution in compound 4 does not improve the plateau reduction, while the 3-nitro substitution in compound 5 demonstrates a plateau reduction of 39.1% ± 19.2%. In addition, the cyclic imide with no fused aromatic rings (compound 1) exhibits a lag extension of 2.8 ± 0.6-fold. This mode of inhibitory capability is lost with the addition of fused unsubstituted aromatic rings (compounds 2 and 3). However, some extension of the lag is regained with the addition of a 3-nitro group (compound 5). Since the 1,8-naphthalimide derivatives (compound 3 and 6) demonstrate the most potent plateau reduction among these series of compounds, the 1,8-naphthalimide group is favorable in these ranitidine analogs to inhibit Aβ aggregation.

### 2.2. Synthesis of Ranitidine Analogs Containing 1,8-Naphthalimide and Their Inhibitory Activities toward Aβ Aggregation and AChE

**Scheme 1:**
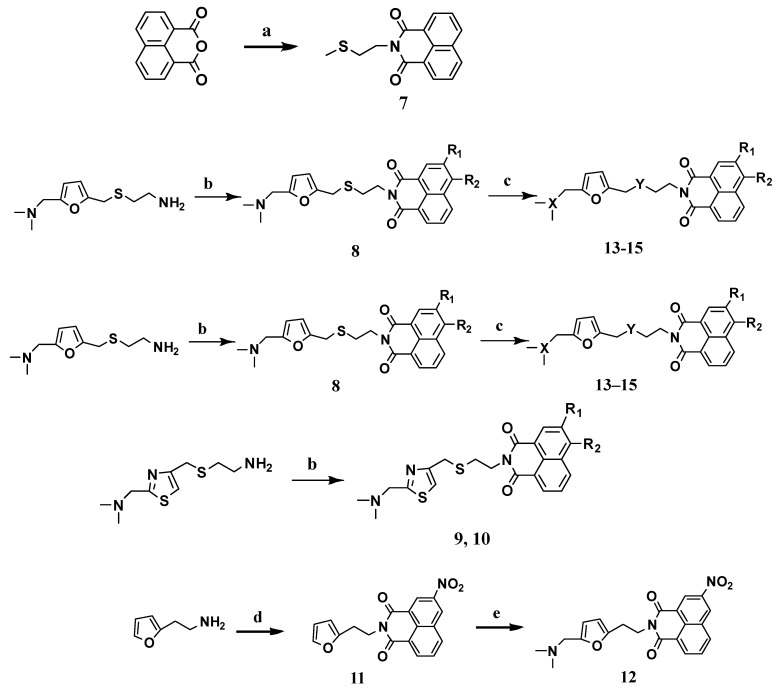
Preparation of ranitidine analogs containing 1,8-naphthalimide (**a**) 2-(Methylthio)ethylamine, toluene, reflux. 5 hrs. (**b**) Correspondent 1,8-naphthalic anhydrides, toluene, reflux. 3–5 hrs. (**c**) H_2_O_2_, CH_3_CH_2_OH, room temperature. overnight, (**d**) 3-nitro-1,8-naphthalic anhydrides, toluene, reflux. 3–5 hrs. (**e**) NH(CH_3_)_2_•HCl, 37% HCHO, 90 °C, 2.5 h.

**Figure 3:**
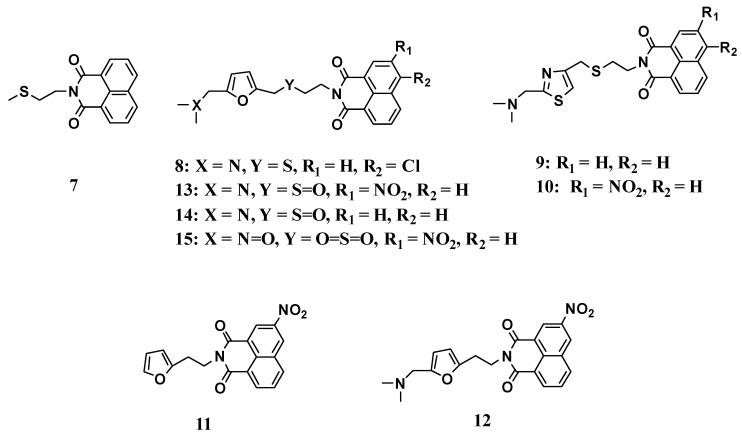
The structure of ranitidine analogs containing 1,8-naphthalimide.

Additional ranitidine analogs containing 1,8-naphthalimide were designed and synthesized through Scheme 1. Their inhibitory activities toward Aβ aggregation are shown in Figure 3, Table 2. All these 1,8-naphthalimide compounds exhibited potent plateau reduction except compound **7**, which lacks one aromatic end group, the inhibitory capacity of which was abrogated. This observation demonstrates that the presence of two aromatic end groups in a molecule is critical for potent Aβ aggregation inhibition. It is also interesting that compounds **9** and **10** with thiazole rings at the other end of the structures demonstrated similar plateau reductions (61.4% ± 19.7% and 54.2% ± 5.1%, respectively) to compounds **3** and **6**, which indicates that the furan rings in these 1,8-naphthalimide-derived ranitidine analogs could be replaced by other aromatic rings while keeping similar Aβ aggregation inhibition. As for the substituents on the 1,8-naphthalimide, the plateau reduction of compound **8** with a 4-chloro substituent is between the reductions of the unsubstituted derivative (compound **3**) and the 3-nitro derivative (compound **6**), which suggested that substituents on the naphthalimide group may enhance the plateau reductions.

Compound **11** was further designed and synthesized to improve the lag time extension of these ranitidine analogs since a two-carbon chain in the 1,8-naphthalimide analogs possesses the optimal length for Aβ aggregation inhibition [14]. As expected, this compound provided a significant plateau reduction of 60.3% ± 9.8% and extended the lag time by 1.76 ± 0.61-fold over the control sample. 

It should also be noted that compound **6** is a potent AChE inhibitor (IC_50_ = 0.15 µM) due to the structural elements of dimethylamine and naphthalimide [11]. In order to revive the AChE inhibition for compound **11**, the dimethyl amino group was added to its structure, leading to compound **12**. Compounds **8**–**10** and **12** were selected for the evaluation of AChE inhibition. As shown in Table 2, these selected compounds have potent inhibitory activities toward AChE. In particular, compound **12** possesses a high efficacy as an AChE inhibitor (IC_50_ = 0.36 µM). Since both Aβ aggregation and AChE are therapeutic targets for AD, these ranitidine analogs could represent a new type of multi-target directed ligands beneficial to the therapy of AD. 

**Figure 4:**
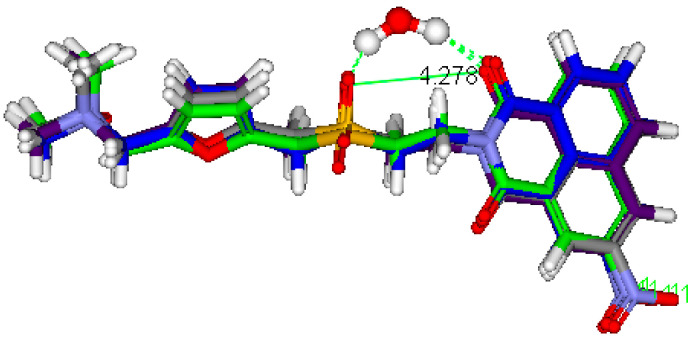
The alignment of 1,8-naphthalimide derivatives in the most energetically favorable computed conformations.

However, the oxidation of sulfur (compounds **13**–**15**) significantly impaired the inhibitory activities of these molecules, which may be due to the rigid conformations of the sulfoxide or sulfone molecules. As proposed in the ideal structure of Aβ aggregation inhibitors, a flexible linker is critical for the conformational compatibility to Phe19-Phe20 in the Aβ peptide in order to facilitate binding to Aβ and interfere with the addition of Aβ monomers to growing aggregates [5]. However, water molecules in the solvents might display a solvation effect via multiple hydrogen bonding interactions and stabilize the conformations of these molecules. As shown in Figure 4, compounds **13**–**15** in their most energetically favorable computed conformations were aligned together with the help of the Discovery Studio (Dassault Systemes, Waltham, MA) [15], a software application that simulates small molecule and macromolecule systems. The length between the oxygen atoms of sulfoxides or sulfones and the oxygen atoms of the imide groups is only 4.278 Å, which is close enough to have hydrogen bonds with one water molecule. These hydrogen bonds could lock the conformation and impede the interactions of these molecules with Aβ peptides. 

## 3. Materials and Methods

### 3.1. Materials

Aβ_40_ was purchased from AnaSpec (AnaSpec, Fremont, CA, USA) and stored in lyophilized form at −20 °C. Thioflavin T (ThT) and electric eel AChE were obtained from Sigma (Sigma-Aldrich, St. Louis, MO, USA). Bovine serum albumin (BSA) and dimethyl sulfoxide (DMSO) were purchased from EMD Biosciences (EMD Biosciences, San Diego, CA, USA). Tris-HCl (pH 8.0) was prepared using Tris base, purchased from Promega (Promega, Madison, WI, USA) and Tris hydrochloride, purchased from EMD (EMD, Burlington, MA, USA). 

### 3.2. Chemical Synthesis

Melting points were determined on an Electrothermal^®^ melting point apparatus (Cole-Parmer, Vermon Hills, IL, USA) and are uncorrected. ^1^H-NMR spectra were obtained on a Mercury VX-300 spectrometer (Varian, Palo Alto, CA, USA), using deuterochloroform (CDCl_3_) as the solvent. Most chemicals were purchased from Acros Organics (Fair Lawn, NJ, USA) and TCI America chemicals (Portland, OR, USA). Mass spectrometry was performed utilizing a VG Analytical 70 S magnetic sector mass spectrometer (Waters, Milford, MA, USA). Chromatography was performed using Geduran^®^ silica gel (40–63 µm).

#### 3.2.1. General Procedure for Compounds **1**–**10**

To a suspension of 1,8-naphthalic anhydride (3.0 g, 15 mmol) in toluene (100 mL), the corresponding reactants (15 mmol) were added dropwise. The reaction mixture was refluxed with a condenser and a Dean–Stark trap for 5 hours and then evaporated to yellow solid, which was recrystallized with toluene (60 mL) to yield products. The structural information of compounds **1**–**6** is available in our previous paper [11].

2-(2-(Methylthio)ethyl)-5-nitro-1H-benzo[de]isoquinoline-1,3(2H)-dione(**7**) Melting point (mp): 153–154 °C; 1H NMR (300 MHz, CDCl_3_) δ 2.19 (s, 3H, SCH_3_), 2.82 (t, J = 7.1, 2H, SCH_2_CH_2_), 4.38 (t, J = 7.1, 2H, CH_2_CH_2_NH), 7.89 (t, J = 7.5, 1H, ArH), 8.8 (d, J = 7.5, 1H, ArH), 8.72 (d, J = 7.5, 1H, ArH), 9.08 (d, J = 7.5, 1H, ArH), 9.27 (d, J = 7.5, 1H, ArH). HRMS: Obs. M+H, 316.0519. Calc. M+H, 316.0518. 

6-Chloro-2-(2-(((5-((dimethylamino)methyl)furan-2-yl)methyl)thio)ethyl)-1H-benzo [de]isoquinoline-1,3(2H)-dione(**8**) mp: 98–99 °C; 1H NMR (300 MHz, CDCl_3_) δ 2.26 (s, 6H, N(CH_3_)_2_), 2.84 (t, J = 7.1, 2H, SCH_2_CH_2_), 3.45 (s, 2H, CCH_2_S), 3.84 (s, 2H, (CH_3_)_2_NCH_2_), 4.37 (t, J = 7.1, 2H, CH_2_CH_2_N), 6.11(d, J = 7.5, 1H, ArH), 6.25 (d, J = 7.5, 1H, ArH), 7.40 (m, 2H, ArH), 8.48 (d, J = 7.5, 1H, ArH), 8.63 (d, J = 7.5, 1H, ArH), 8.65 (d, J = 7.5, 1H, ArH). HRMS: Obs. M+H, 429.1039. Calc. M+H, 429.1039.

2-(2-((2-((Dimethylamino)methyl)thiazol-4-yl)methylthio)ethyl)-1H-benzo[de]iso quinoline-1,3(2H)-dione (**9**) mp: 116–118 °C; 1H NMR (300 MHz, CDCl_3_) δ 2.35 (s, 6H, N(CH_3_)_2_), 2.83 (t, J = 7.1, 2H, SCH_2_CH_2_), 3.77 (s, 2H, CCH_2_S), 3.97 (s, 2H, (CH_3_)_2_NCH_2_), 4.42(t, J = 7.1, 2H, CH_2_CH_2_NH), 7.28 (s, 1H, ArH), 7.77 (t, J = 7.5, 2H, ArH), 8.23 (d, J = 7.5, 2H, ArH), 8.62 (d, J = 7.5, 2H, ArH). HRMS: Obs. M+H, 412.1152. Calc. M+H, 412.1153. 

2-(2-(((2-((Dimethylamino)methyl)thiazol-4-yl)methyl)thio)ethyl)-5-nitro-1H-benzo[de]isoquinoline-1,3(2H)-dione (**10**) mp: 124–125 °C; 1H NMR (300 MHz, CDCl3) δ 2.36 (s, 6H, N(CH_3_)_2_), 2.84 (t, J = 7.1, 2H, SCH_2_CH_2_), 3.78 (s, 2H, CCH_2_S), 3.96 (s, 2H, (CH3)_2_NCH_2_), 4.44 (t, J = 7.1, 2H, CH_2_CH_2_NH), 7.29 (s, 1H, ArH), 8.23 (t, J = 7.5, 1H, ArH), 8.28 (d, J = 7.5, 1H, ArH), 8.82 (d, J = 7.5, 1H, ArH), 8.87 (d, J = 7.5, 1H, ArH), 9.35 (d, J = 1.5, 1H, ArH). HRMS: Obs. M+H, 456.5409. Calc. M+H, 456. 5409.

#### 3.2.2. General Procedure for Compound **12**

To a suspension of 3-nitro-1,8-naphthalic anhydride (4 mmol) in toluene (50 mL), a solution of 2-furan-2-yl-ethanolamine (4 mmol) in ethanol was added dropwise. The reaction mixture was refluxed with a condenser and a Dean–Stark trap for 6 hours and then was evaporated under reduced pressure. The resulting residue was purified by column chromatography. The silica gel was first conditioned with dichloromethane, and then the product was eluted with 50:1 dichloromethane/methanol. Evaporation of the solvent yielded compound **11**. Dimethylamine hydrochloride (1.2 mmol) and compound **11** (0.6 mmol) were added to a stirred 37% formaldehyde solution (0.12 mol) under a nitrogen atmosphere. The solution was heated from 25 °C to 90 °C for 20 hours and then was allowed to cool to room temperature, transferred to a separatory funnel, and then extracted with 20 mL of dichloromethane. The aqueous layer was stirred with a Na_2_CO_3_-saturated solution (20 ml). The mixture was extracted with dichloromethane (10 mL for three times. The organic solution was washed with distilled water, dried over MgSO_4_, and concentrated to dryness. The resulting residue was purified by vacuum distillation to yield compound **12**. 

2-(2-(5-((Dimethylamino)methyl)furan-2-yl)ethyl)-5-nitro-1H-benzo[de]isoquino line-1,3(2H)-dione (**11**) mp: 85–87 °C; 1H NMR(300MHz, CDCl_3_) δ 2.24 (s, 6H, N(CH_3_)_2_, δ 3.08(t, J = 7.1, 2H, CH_2_CH_2_N), 3.39(s, 2H, (CH_3_)_2_NCH_2_), 4.49(t, J = 7.1, 2H, CH_2_CH_2_N), 5.99(d, J = 7.5, 1H, ArH), 6.06(t, J = 7.5, 1H, ArH), 7.94(t, J = 7.5, 1H, ArH), 8.43(d, J = 7.5, 1H, ArH), 8.76(d, J = 7.5, 1H, ArH), 9.13(d, J = 1.5, 1H, ArH), 9.29(d, J = 1.5, 1H, ArH). HRMS: Obs. M+H, 394.1404. Calc. M+H, 394.1403. 

2-(2-(furan-2-yl)ethyl)-5-nitro-1H-benzo[de]isoquinoline-1,3(2H)-dione (**12**) mp:182–183 °C; 1H NMR(300MHz, CDCl_3_) δ 3.10(t, J = 7.1, 2H, CH_2_CH_2_N), 4.49(t, J = 7.1, 2H, CH_2_CH_2_N), 6.09(d, J = 7.5, 1H, ArH), 6.83(t, J = 7.5, 1H, ArH), 7.31(d, J = 7.5, 1H, ArH), 7.95(t, J = 7.5, 1H, ArH), 8.82(d, J = 7.5, 1H, ArH), 8.77(d, J = 7.5, 1H, ArH), 9.14(d, J = 1.5, 1H, ArH), 9.31(d, J = 1.5, 1H, ArH). HRMS: Obs. M+H, 336.0747. Calc. M+H, 336.0746.

#### 3.2.3. General Procedure for Compounds **13**–**15**

A solution of compound **6** (0.5 mmol) in glacial acetic acid (20 mL) was treated with 35% hydrogen peroxide (38 µL) in ice bath. The mixture was stirred while warming to room temperature overnight and then was evaporated under reduced pressure. The resulting residue was purified by flash column chromatography using dichloromethane/methanol mobile system. Evaporation of the solvent yielded products compound **13** and **15**. Compound **14** was synthesized from compound **3** using the same procedure.

2-(2-(((5-((Dimethylamino)methyl)furan-2-yl)methyl)sulfinyl)ethyl)-5-nitro-1H-benzo[de]isoquinoline-1,3(2H)-dione(**13**) mp: 106.5–108 °C; 1H NMR (300 MHz, CDCl_3_) δ 2.28 (s, 6H, N(CH_3_)_2_), 3.10 (t, J = 7.1, 2H, SCH_2_CH_2_), 3.51 (s, 2H, CCH_2_S), 4.17 (s, 2H, (CH_3_)_2_NCH_2_), 4.68 (t, J = 7.1, 2H, CH_2_CH_2_N), 6.24 (d, J = 7.5, 1H, ArH), 6.39 (d, J = 7.5, 1H, ArH), 7.95 (t, J = 7.5, 1H, ArH), 8.45 (d, J = 7.5, 1H, ArH), 8.80 (d, J = 7.5, 1H, ArH), 9.16 (d, J = 1.5, 1H, NO_2_ArH), 9.34 (d, J = 1.5, 1H, NO_2_ArH). HRMS: Obs. M+H, 456.1232. Calc. M+H, 456.1229.

2-(2-(((5-((Dimethylamino)methyl)furan-2-yl)methyl)sulfinyl)ethyl)-1H-benzo[de] isoquinoline-1,3(2H)-dione(**14**) mp: 117–119 °C; 1H NMR (300 MHz, CDCl_3_) δ 2.22 (s, 6H, N(CH_3_)_2_), 3.09 (t, J = 7.1, 2H, SCH_2_CH_2_), 3.43 (s, 2H, CCH_2_S), 4.18 (s, 2H, (CH_3_)_2_NCH_2_), 4.64 (t, J = 7.1, 2H, CH_2_CH_2_N), 6.19 (d, J = 7.5, 1H, ArH), 6.39 (d, J = 7.5, 1H, ArH), 7.76 (t, J = 7.5, 2H, ArH), 8.24 (d, J = 7.5, 2H, ArH), 8.62 (d, J = 7.5, 1H, ArH). HRMS: Obs. M+H, 411.1382. Calc. M+H, 411.1378. 

N,N-Dimethyl-1-(5-(((2-(5-nitro-1,3-dioxo-1H-benzo[de]isoquinolin-2(3H)-yl)ethyl) sulfonyl)methyl)furan-2-yl)methanamine oxide(**15**) mp: 138–40 °C; 1H NMR (300 MHz, CDCl_3_) δ 3.18 (s, 6H, N(CH_3_)_2_), 3.47 (t, 2H, SO_2_CH_2_CH_2_), 4.47 (s, 2H, (CH_3_)_2_NCH_2_), 4.58 (s, 2H, CCH_2_SO_2_), 4.71 (t, J = 7.1, 2H, CH_2_CH_2_N), 6.69 (s, 2H, ArH), 7.98 (t, J = 7.5, 1H, ArH), 8.47 (d, J = 7.5, 1H, ArH), 8.82 (d, J = 7.5, 1H, ArH), 9.17 (d, J = 7.5, 1H, ArH), 9.34 (d, J = 7.5, 1H, ArH). HRMS: Obs. M+H, 488.1141. Calc. M+H, 488.1127.

### 3.3. Biological Assays

#### 3.3.1. Preparation of Aβ Monomers

Lyophilized Aβ peptide was reconstituted in 50 mM NaOH at a concentration of 2 mg/mL and purified via size-exclusion chromatography (SEC) on a Superdex 75 HR 10/300 column (Cytiva, Marlborough, MA) using a running buffer of 40 mM Tris-HCl (pH 8.0). Bovine serum albumin (BSA) (2 mg/mL) was employed as a pretreatment to reduce nonspecific Aβ interaction with the dextran matrix of the column. The elution profile showed a characteristic peak corresponding to monomeric Aβ. The concentrations of eluted Aβ monomer fractions were determined with a calculated extinction coefficient of 1450 M^−1^ cm^−1^ at 276 nm. Purified Aβ monomer samples were used fresh or stored at 4 ℃ for up to 4 days.

#### 3.3.2. Preparation of Ranitidine Analogs

All compounds were dissolved in DMSO at 5 mM and stored at −20 °C. Immediately prior to experimentation, stock solutions were diluted into DMSO such that subsequent dilution to 100 μM ranitidine analog yielded 2% (*v*/*v*) DMSO in the final reaction solution. DMSO (2% *v*/*v*) was added to control reactions run in parallel.

#### 3.3.3. ThT Fluorescence Assay

SEC-purified Aβ monomer was diluted to 40 μM in 40 mM Tris-HCl (pH 8.0) and agitated (vortex, 800 rpm, 25 °C) without (control) or with 100 µM ranitidine analogs. Periodically, a 20-μL aliquot was removed and combined with 140 μL of 10 μM ThT. Fluorescence (excitation = 450 nm, emission = 470–500 nm) was evaluated using a Perkin-Elmer LS-45 luminescence spectrometer (Waltham, MA, USA). Fluorescence values were calculated as the integrated area under the emission curve with baseline (ThT alone) subtraction and plotted vs. aggregation time. Each compound was evaluated using at least three independent experiments. 

#### 3.3.4. AChE Inhibition Assay 

The AChE inhibition assay was modified based on the method of Ellman et al. [16] in a 24-well plate format at 37 °C. Assays were performed in 0.1 M sodium phosphate (pH 8.0), containing 200 µM acetylthiocholine, 100 µM dithiobisnitrobenzoic acid, and 0.005 units of AChE in a final volume of 3000 µL. The reaction was initiated by the addition of acetylthiocholine, and the formation of a product was monitored by measuring absorbance at 412 nm. The IC_50_ values were determined by nonlinear regression analysis of the concentration–response curves generated. Each compound was evaluated using at least three independent experiments.

## 4. Conclusions

In this study, a series of multi-target ranitidine analogs containing cyclic imide isosteres was explored through rationally designed structural modifications. Of particular note, the analogs with 1,8-naphthamide resulted in potent inhibition of Aβ aggregation. While improving the Aβ aggregation inhibition, these structural modifications also retained high efficacy as AChE inhibitors. These results imply that this series of ranitidine analogs represents a new type of MTDLs with inhibitory activities of both Aβ aggregation and AChE. The structural elements in these analogs can be further explored to develop more effective MTDLs for the pharmaceutical treatment of AD.

## Figures and Tables

**Table 1:** The Aβ monomer aggregation inhibitory capability of compounds **1**–**6** ^a^.

Compound	Plateau Reduction (% Inhibition)	Lag Time Extension (Fold Increase)
**1**	N.E.	2.8 ± 0.6
**2**	N.E.	N.E.
**3**	60.9% ± 5.7%	N.E.
**4**	N.E.	N.E.
**5**	39.1% ± 19.2%	2.0 ± 0.6
**6**	71.8% ± 3.9%	N.E.

The observed plateau or lag time in the presence of 100 µM compound is not significantly different from that observed for control samples. ^a^ Inhibitions are expressed as mean ± SEM, *n* = 3–5.

**Table 2:** The inhibition capabilities of ranitidine analogs containing 1,8-naphthalimide on Aβ monomer aggregation ^a^ and AChE activity.

Compound	Plateau Reduction (% Inhibition)	Lag Time Extension (Fold Increase)	AChE (IC_50_ µM)
**7**	N.E.	N.E.	N.T.
**8**	65.8% ± 3.5%	N.E.	1.23
**9**	54.2% ± 5.1%	N.E.	2.02
**10**	61.4% ± 19.7%	N.E.	0.52
**11**	60.3% ± 9.8%	1.76 ± 0.61	N.T.
**12**	65.6% ± 4.1%	2.25 ± 0.25	0.36
**13**	26.8% ± 10.4%	N.E.	N.T.
**14**	20.1% ± 4.6%	N.E.	N.T.
**15**	20.4% ± 9.6%	N.E.	N.T.

The observed plateau or lag time in the presence of 100 µM compounds is not significantly different from that observed for control samples. N.T.: not tested. ^a^ Inhibitions are expressed as mean ± SEM, *n* = 3–5.
